# Mental health and cognitive function among medical students after the COVID-19 pandemic in China

**DOI:** 10.3389/fpubh.2023.1233975

**Published:** 2023-07-28

**Authors:** Junzhe Cheng, Mei Liao, Ziping He, Rui Xiong, Yumeng Ju, Jin Liu, Bangshan Liu, Bei Wu, Yan Zhang

**Affiliations:** ^1^Department of Psychiatry, National Clinical Research Center for Mental Disorders, and National Center for Mental Disorders, The Second Xiangya Hospital of Central South University, Changsha, Hunan, China; ^2^Clinical Medicine Eight-Year Program, Xiangya School of Medicine, Central South University, Changsha, Hunan, China; ^3^Mental Health Institute of Central South University, China National Technology Institute on Mental Disorders, Hunan Key Laboratory of Psychiatry and Mental Health, Hunan Medical Center for Mental Health, Changsha, Hunan, China; ^4^School of Stomatology, Nanchang University, Nanchang, Jiangxi, China; ^5^Hospital Management Office, Central South University, Changsha, Hunan, China

**Keywords:** medical students, post-COVID-19 period, mental health, cognitive function, risk factors

## Abstract

**Background:**

Chinese people experienced a nationwide coronavirus disease 2019 (COVID-19) pandemic after the adjustment of epidemic response policies from December 2022 to January 2023. This study aims to explore the prevalence of mental and cognitive symptoms and their associated factors among medical students after the COVID-19 pandemic.

**Methods:**

A cross-sectional study was conducted between February 27th and March 8th, 2023. The symptoms of anxiety, depression, insomnia, post-traumatic stress disorder (PTSD), and cognitive function among medical students were examined using the Generalized Anxiety Disorder-7 (GAD-7), the Patient Health Questionnaire-9 (PHQ-9), the Athens Insomnia Scale (AIS), the Impact of Event Scale-6 (IES-6), and the Perceived Deficits Questionnaire-Depression-5 (PDQ-D-5). Data on demographic information was also collected. Statistical analyses were conducted to describe the prevalence and explore the associated factors of mental and cognitive symptoms.

**Results:**

Among 947 participants, the proportion of students experiencing anxiety, depression, insomnia, and PTSD symptoms was 37.8, 39.3, 28.3, and 29.5%, respectively. The self-reported COVID-19 infection rate was 72.2%. Higher grades, childhood, and current rural residence were identified as potential risk factors for mental and cognitive symptoms. Gender, age, average monthly household income, and COVID-19 diagnosis were not associated with mental and cognitive symptoms among medical students.

**Conclusion:**

Our findings revealed a high prevalence of mental and cognitive symptoms among Chinese medical students after the COVID-19 pandemic. Special attention should be paid to the mental health of higher-grade students and those residing in rural areas.

## 1. Introduction

With the alterations of the Chinese epidemic prevention policy, China experienced a nationwide wave of the coronavirus disease 2019 (COVID-19) pandemic from December 2022 to January 2023. The Chinese Center for Disease Control and Prevention (CDC) reported a 6.94 million peak in the number of infected people (1). According to reports, the general public’s physical and mental health has been adversely affected by the COVID-19 epidemic (2, 3). To embark on targeted therapies after the COVID-19 pandemic, it is imperative to emphasize people’s mental health and identify the susceptible population. Compared to other social populations, college students exhibited a higher vulnerability to the COVID-19 outbreak, encountering uncertainty and unexpected disruptions to their academic semesters (4). The COVID-19 pandemic and subsequent quarantine measures gave rise to various outcomes for college students (5). They found it challenging academically to transition from traditional face-to-face learning to online learning (6, 7). Long-term isolation at home and minimal social interaction may contribute to mental disorders, including depression and anxiety (8).

Medical students were considered a high-risk group for suffering from mental disorders (9). The mental health of medical students had already started to deteriorate prior to the COVID-19 pandemic (10–12). Some of them even actively participated in efforts to provide COVID-19 aid during the pandemic (11, 13). Additionally, lockdown measures including digital learning, home quarantine, and social distancing were unprecedented experiences for them. A cross-sectional survey conducted on medical students in China showed that the outbreak of COVID-19 aggravated medical students’ negative emotional outcomes (14). During the long-term pandemic, medical students tended to suffer from several cognitive symptoms and mental disorders, such as anxiety, depression, insomnia, post-traumatic stress disorder (PTSD), and cognitive dysfunction (15–17). The impaired mental health and cognitive function may negatively impact the future professional identity and capacity to care for patients. Examining the extent of physical and psychological well-being in the post-epidemic era is crucial for ensuring the future quality of healthcare services (18).

Medical students were considered as a high-risk group for mental disorders (9). The mental health of medical students had already started to deteriorate prior to the COVID-19 pandemic (10–12). Some medical students even actively participated in efforts to provide COVID-19 aid during the pandemic (11, 13). Additionally, lockdown-associated changes including digital learning, home quarantine, and social distancing were unprecedented experiences for them. A cross-sectional survey conducted on medical students in China showed that the outbreak of COVID-19 aggravated medical students’ negative emotional outcomes (14). During the long-term pandemic, medical students tended to suffer from several cognitive symptoms and mental disorders, such as anxiety, depression, insomnia, post-traumatic stress disorder (PTSD), and cognitive dysfunction (15–17). The impaired mental health and cognitive function may negatively impact their enthusiasm and capacity to care for patients. Examining the level of physical and psychological well-being of medical students in the post-epidemic era is crucial for ensuring the quality of healthcare services in the future (18).

Previous studies primarily investigated the physical and mental well-being of college students during the early stage of the pandemic from 2020 to 2022 (19, 20). Although extensive research has been conducted on the initial phases of the epidemic, inadequate effort has been emphasized for medical students in the post-COVID-19 period. However, most of the students who were infected during the first wave of the COVID-19 pandemic in China were psychologically prepared for the subsequent infection, given their prior experience. As a result, they may perceive themselves well-informed about the pandemic during this period, which might reduce their anxiety and fear (21, 22). Moreover, residence (urban or rural) and family income stability are also associated with psychological well-being (23). In response, we conducted a survey on Chinese medical students to investigate the prevalence and severity of mental and cognitive symptoms and their associated factors, and to provide validated empirical data on mental health and cognitive function after the COVID-19 pandemic. Our study was expected to enrich the evidence of mental health and cognitive function among medical students in the post-COVID-19 period and provide clues for identifying a targeted population for psychological intervention.

## 2. Methods

### 2.1. Participants and study design

A cross-sectional survey was conducted offline using nonprobability sampling among medical students. It started on February 27th, 2023, and ended on March 8th, 2023, after China experienced the first round of the COVID-19 pandemic nationwide. The survey was administered offline using paper questionnaires at Central South University, Changsha, Hunan, China. A total of 1,049 medical students participated in the survey. Every student who followed the survey gave informed consent about the research protocol. Participants who met the following criteria were included: (1) medical students, (2) students studying at Central South University, and (3) volunteered for the survey. The exclusion criteria included missing or inadequate information and/or any major physical or mental illness. Ultimately, 947 medical students were included in the study.

### 2.2. Ethical considerations

This study had been approved by the Ethics Committee of the Second Xiangya Hospital of Central South University (approval number: 047).

### 2.3. Measurements

#### 2.3.1. Demographic information

Participants provided demographic information on their age, gender, grade, average monthly household income, childhood residence, current residence, history of physical illness, psychiatric history, and whether they had been diagnosed with COVID-19 infection.

#### 2.3.2. Anxiety symptoms

The seven-item General Anxiety Disorder-7 (GAD-7) was used to record anxiety symptoms (24). Participants rated the frequency of experiencing seven symptoms during the previous 2 weeks as: (0) not at all, (1) on several days, (2) on more than half of the days, and (3) nearly every day. Scores range from 0 to 21, with higher scores indicating more anxiety symptoms. The total score *S* of GAD-7 is categorized as follows: *S* ≤ 4 = minimal symptoms; 5 ≤ *S* ≤ 9 = mild symptoms; 10 ≤ *S* ≤ 13 = moderate symptoms; 14 ≤ *S* ≤ 18 = moderately severe symptoms; *S* ≥ 19 = severe symptoms. With a Cronbach’s alpha of 0.879, the GAD-7 in the current sample shows a strong level of internal consistency.

#### 2.3.3. Depressive symptoms

Depressive symptoms were assessed using the nine-item Patient Health Questionnaire (PHQ-9) (25, 26). A Likert scale (0 = “Not at all” to 3 = “Nearly every day”) was used to assess the frequency of experiencing nine symptoms during the previous 2 weeks. Scores range from 0 to 27, with higher scores indicating more depressive symptoms. The total score *S* of PHQ-9 is categorized as follows: *S* ≤ 4 = minimal symptoms; 5 ≤ *S* ≤ 9 = mild symptoms; 10 ≤ *S* ≤ 14 = moderate symptoms; 15 ≤ *S* ≤ 19 = moderately severe symptoms; *S* ≥ 20 = severe symptoms. The PHQ-9 in the current sample has shown an internal consistency of 0.908 (Cronbach’s alpha).

#### 2.3.4. Insomnia

The eight-item Athens Insomnia Scale (AIS) was used to record the severity of insomnia for all subjects (27). A Likert scale including five items related to sleep difficulties and three related to daytime functional impairment (0 = “Not at all” to 3 = “Extremely”) was used to assess the severity during the past month. The total score ranges from 0 to 24 points. Previous studies have confirmed that a score of 6 is a reasonable cutoff for insomnia (28). On this basis, an AIS score < 4 was defined as normal, a 4 ≤ score < 6 was defined as suspected insomnia, and a score ≥ 6 was defined as insomnia. The AIS in the current sample has internal consistency (Cronbach’s alpha, 0.805).

#### 2.3.5. PTSD symptoms

The level of PTSD is usually measured by the Impact of Event Scale-6 (IES-6) for research in epidemiological studies or clinical practice. IES-6 is validated and shortened based on the Impact of Events Scale-Revised (IES-R) (29). Participants were asked to report their PTSD symptoms in the past 7 days on six items from 0 (not at all) to 4 (extremely). Scores range from 0 to 24, with higher scores indicating more PTSD symptoms. It can associate the COVID-19 pandemic with PTSD symptoms through its three subscales (intrusion, avoidance, and hyperarousal). We used the mean score *S* of IES-6 to measure the level of PTSD: *S* < 1.09 = normal; 1.09 ≤ *S* < 1.5 = showing PTSD symptoms; *S* ≥ 1.5 = may be diagnosed with probable PTSD (30). The IES-6 in our study sample showed an internal consistency of 0.862 (Cronbach’s alpha).

#### 2.3.6. Cognitive dysfunctions

The five-item Perceived Deficits Questionnaire-Depression (PDQ-D-5) was used to record the severity of self-reported cognitive symptoms over the previous 7 days (31). The frequency of experiencing these symptoms is rated using a scale ranging from 0 to 4 (0 = “not at all” to 3 = “nearly every day”). The total score ranges from 0 to 20, with higher scores indicative of more severe cognitive symptoms. With a Cronbach’s alpha of 0.852, the PDQ-D-5 in the current sample exhibits a favorable score for internal consistency.

### 2.4. Statistical analysis

Data were analyzed using SPSS Version 25.0 (IBM SPSS, Armonk NY, USA). Descriptive statistical analyses were conduct to exhibit the sample’s demographic profile and level of mental health and cognitive function. Reliability tests were used to check the internal consistency of IES-6, GAD-7, PHQ-9, AIS, and PDQ-D-5 in the current sample. Based on the data type and data distribution, independent t-tests, person correlation analysis and spearman correlation analysis were respectively adopted to examine the relationships between participants’ demographics and mental health, sleep quality, and cognitive function. A two-tailed *p*<0.05 was considered statistically significant.

## 3. Results

### 3.1. Demographic characteristics

The general demographic characteristics of these 947 medical students are shown in Table 1. The final sample of participants for the present study consisted of 947 medical students, with a median age of 18.88 (SD: 0.23) years and the majority of participants between the ages of 15 and 24. Four hundred and fifty-nine (48.5%) male and five hundred and twelve (51.5%) female participants responded to the survey. The sample of medical students consists of 521 freshmen (55.0%) and 426 sophomores (45.0%). Most participants had an average monthly household income of <10,000 yuan (81.1%). Most participants had the urban census register in childhood (65.2%), and the ratio has increased to 82.2% at present. Most participants reported a history of a positive diagnosis of COVID-19 (72.2%).

**Table 1:** Demographic characteristics of the responders (*n* = 947).

Variables		
Continuous variables		*Means* ± *SD*
Age (years)		18.88 ± 0.923
Categorical variables		*No. (%)*
Gender	Male	459 (48.5)
	Female	512 (51.5)
Grade	Freshman	521 (55.0)
	Sophomore	426 (45.0)
Average monthly household income	<1,000	38 (4.0)
	1,000–5,000	438 (46.3)
	5,000–10,000	292 (30.8)
	10,000–50,000	167 (17.6)
	>50,000	12 (1.3)
Childhood residence	Rural	310 (32.7)
	Urban	617 (65.2)
	Prefer to no answer	20 (2.1)
Current residence	Rural	145 (15.3)
	Urban	778 (82.2)
	Prefer to no answer	24 (2.5)
Diagnosed with Covid-19?	Yes	684 (72.2)
	No	170 (18.0)
	Not sure	93 (9.8)

SD: Stand deviation.

### 3.2. The prevalence of anxiety, depression, insomnia, and PTSD symptoms

**Figure 1:**
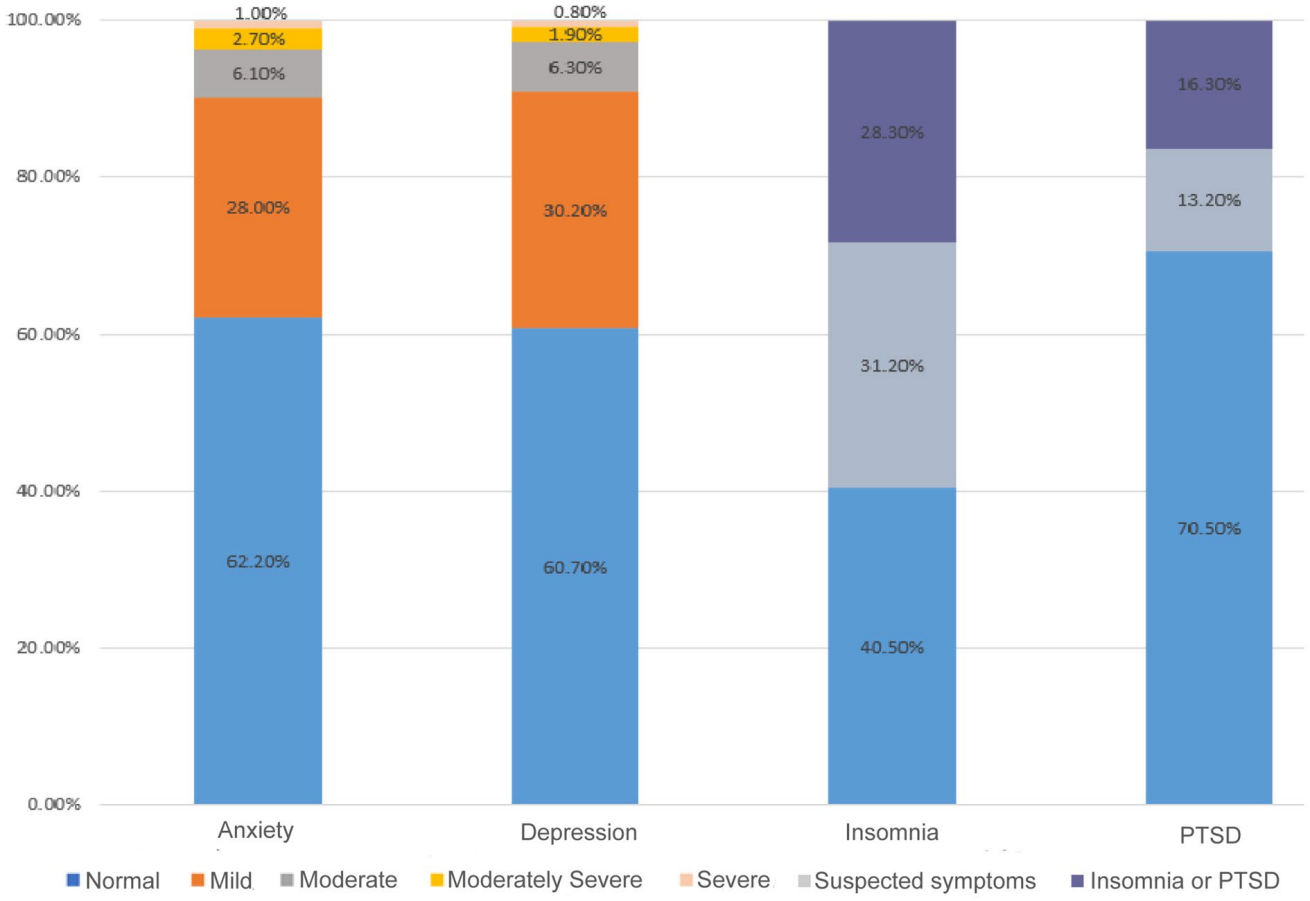
The prevalence of anxiety, depression, insomnia, and PTSD symptoms among medical students. PTSD: post-traumatic stress disorder.

Overall, the proportion of participants reporting mild, moderate, moderately severe, and severe anxiety symptoms was 28.0, 6.1, 2.7, and 1.0%, respectively. The proportion of participants reporting mild, moderate, moderately severe, and severe depressive symptoms was 30.2, 6.3, 1.9, and 0.8%, respectively. The proportions of participants reporting suspected insomnia and insomnia were 31.2 and 28.3%, respectively. Whereas, the proportions of participants reporting PTSD symptoms and probable PTSD were 13.2 and 16.3%, respectively (Figure 1).

### 3.3. Factors associated with mental and cognitive symptoms

Table 2 displays the factors that may be associated with mental and cognitive symptoms. There were no significant gender differences in either mental health or cognitive function. It was found that compared to freshmen, sophomores had higher levels of anxiety symptoms (*p* = 0.03), depressive symptoms (*p <* 0.01), insomnia (*p <* 0.01), and PTSD symptoms (*p* < 0.01). Moreover, there was no significant difference in cognitive symptoms between different grades. The rural students showed significantly higher cognitive symptoms both in their childhood residence (*p* < 0.01) and current residence (*p* = 0.03) than the urban students. However, there was no significant difference in mental health and cognitive function whether the participant was diagnosed with COVID-19 or not.

**Table 2:** Independent *t*-tests of sociodemographic characteristics and mental health of medical students.

Variables			GAD-7 scores	PHQ-9 scores	AIS scores	IES-6 scores	PDQ-D scores
Gender	Male	Mean (S.D.)	4.08 (4.38)	4.19 (4.38)	4.82 (3.75)	4.58 (4.30)	4.53 (3.80)
	Female		4.13 (3.71)	4.20 (4.04)	4.71 (3.41)	4.41 (3.81)	4.80 (3.85)
		T	−0.17	−0.03	−0.47	0.65	−1.06
		*p*-value	0.87	0.97	0.64	0.51	0.29
Grade	Freshman	Mean (S.D.)	3.86 (3.89)	3.87 (3.98)	4.03 (3.90)	4.03 (3.90)	4.65 (3.83)
	Sophomore		4.42 (4.22)	4.59 (4.45)	5.06 (4.18)	5.06 (4.18)	4.67 (3.83)
		T	−2.127	−2.624	−3.872	−3.872	−0.063
		*p*-value	**0.03**	**<0.01**	**<0.01**	**<0.01**	0.95
Childhood residence	Rural	Mean (S.D.)	4.12 (3.73)	4.55 (4.04)	4.48 (3.98)	4.48 (3.98)	5.24 (3.76)
	Urban		4.13 (4.22)	4.05 (4.31)	4.52 (4.11)	4.52 (4.11)	4.39 (3.83)
		T	−0.038	1.718	−0.139	−0.139	3.233
		*p*-value	0.97	0.09	0.89	0.89	**<0.01**
Current residence	Rural	Mean (S.D.)	4.14 (3.31)	4.61 (3.65)	4.89 (3.91)	4.89 (3.91)	5.28 (3.55)
	Urban		4.14 (4.20)	4.15 (4.33)	4.44 (4.08)	4.44 (4.08)	4.54 (3.85)
		T	0.027	1.204	1.23	1.23	2.162
		*p*-value	0.98	0.09	0.22	0.22	**0.03**
Diagnosed with Covid-19?	Yes	Mean (S.D).	4.29 (4.19)	4.35 (4.31)	4.63 (4.10)	4.63 (4.10)	4.84 (3.87)
	No		3.92 (3.81)	3.97 (4.00)	4.47 (4.15)	4.47 (4.15)	4.53 (3.72)
		T	1.033	1.049	0.433	0.433	0.941
		*p*-value	0.30	0.29	0.67	0.67	0.35

M: Mean; S.D.: Standard deviation; PHQ-9: Patient Health Questionnaire-9; GAD-7: Generalized Anxiety Disorder-7; AIS: Athens Insomnia Scale; IES-6: Impact of Event Scale-6; PDQ-D: Perceived Deficits Questionnaire-Depression. Bold values indicate statistical significance (*p* < 0.05).

In the correlation analysis of age with mental problems and cognitive symptoms, the grade was considered as a covariate. Childhood and current residence were taken as covariates in the correlation analysis of average monthly household income with mental problems and cognitive symptoms. Table 3 displays the results of the bivariate partial correlation analysis of age, average monthly household income, anxiety, depression, insomnia, PTSD, and cognitive symptoms. There were statistically significant positive correlations between anxiety, depression, insomnia, PTSD, and cognitive symptoms. None of the mental or cognitive symptoms were significantly correlated with age or the average monthly household income.

**Table 3:** Correlation analysis of age, average monthly household income, mental health, and cognitive function of medical students.

		Age	Average monthly household income	Anxiety	Depression	Insomnia	PTSD symptoms	Cognitive symptoms
Age	R	1.00						
	*p*-value							
Average monthly household income	R		1.00					
	*p*-value							
Anxiety	R	−0.05^a^	0.01^b^	1.00				
	*p*-value	0.13	0.96					
Depression	R	0.03^a^	0.01^b^	0.70^c^	1.00			
	*p*-value	0.32	0.79	**0.00**				
Insomnia	R	0.03^a^	−0.01^b^	0.48^c^	0.63^c^	1.00		
	*p*-value	0.38	0.70	**0.00**	**0.00**			
PTSD symptoms	R	0.05^a^	0.04^b^	0.34^c^	0.37^c^	0.33^c^	1.00	
	*p*-value	0.15	0.19	**0.00**	**0.00**	**0.00**		
Cognitive symptoms	R	−0.01^a^	−0.01^b^	0.45^c^	0.59^c^	0.53^c^	0.31^c^	1.00
	*p*-value	0.78	0.79	**0.00**	**0.00**	**0.00**	**0.00**	

Bold values indicate statistical significance (*p* < 0.05).

a Partial correlation analysis with grade controlled.

b Partial correlation analysis with childhood and current residence controlled.

c Pearson analysis; PTSD, post-traumatic stress disorder.

## 4. Discussion

To the best of our knowledge, this is the first study that has examined the mental health and cognitive function of medical students after the first nationwide COVID-19 pandemic in China. Particularly, few studies investigated the cognitive function of medical students during or after the COVID-19 pandemic. The proportions of medical students who experienced symptoms of anxiety, depression, insomnia, or PTSD were 37.8, 39.3, 28.3, and 29.5%, respectively, which were significantly higher than general college students post COVID-19 pandemic (32–34). A cross-sectional study of medical students in Greece during the COVID-19 pandemic reported a higher prevalence of anxiety (67.6%), depression (43.7%), and insomnia (65.9%) than our study (35). It is possible that in our study, with sufficient knowledge about the coronavirus infection and long-term adaptation to the pandemic and quarantine policies, the prevalence of mental and symptoms in medical students faded away to a lower level. Our study showed no significant differences in mental health and cognitive function between male and female students. It might be owing to the similar stresses and negative emotions among male and female medical students as a result of the environment of medical education (23). In addition, sophomores were statistically more likely to experience anxiety, depression, insomnia, and PTSD symptoms than freshmen. It is possible due to increasing pressure from learning, exams, and career choices in higher-grade students.

It is interesting to note that despite receiving a COVID-19 diagnosis, the mental health and cognitive function of the medical students have not changed significantly. Prior investigations have demonstrated that the COVID-19 pandemic’s detrimental repercussions dramatically elevated levels of anxiety and depression (36–38). However, after the long-standing COVID-19 pandemic, the negative impacts, including stigma and fear of COVID-19, progressively subsided in China. Moreover, we speculated that a series of COVID-19-related factors jointly impair the mental health of medical students during the COVID-19 pandemic, including the lockdown measurement, the disruption of clinical training or plans, the excessive learning stress, long screen time due to online learning, less social contact, less exercise, exposure to high-risk environments, and a lack of capacity to handle the unpredictable incident effectively (39–42). The COVID-19 infection is merely one of the factors impairing mental health. Since the COVID-19 pandemic has ended and epidemic policy has been altered, some of the social influences previously mentioned did not pose a threat to medical students anymore.

Medical students with childhood or current rural census registers had significantly higher PDQ-D-5 scores, indicating worse cognitive function compared with urban medical students. Due to the financial, cultural, and educational differences between rural and urban environments, rural medical students may experience an inadaptation to a new life when coming to a university in an urban area (43). The inadaptation may induce concentration and memory decreases, which cause significant differences in PDQ-D-5 scores between rural and urban students. These results indicated the importance of considering the adaptation of rural medical students to the college environment. There was no significant difference between rural and urban students concerning anxiety, depression, insomnia, or PTSD symptoms. The finding of undifferentiated mental health among medical students from rural and urban regions does not corroborate the findings of other rural–urban disparities surveys in China, which generally show that rural college students tend to have worse mental health (depression and anxiety) than urban students (44). However, a prior survey also showed that there was no significant difference in the prevalence of some mental disorders (anxiety, insomnia) between rural and urban students (45). It does not imply that the data collected from regular college students or medical students was “incorrect” or “not replicated.” Rather, these inconsistent findings reveal the importance of considering the differences between medical and nonmedical college students in terms of their respective learning environments, educational cultures, and social expectations.

Some limitations are important to consider in light of the current investigation. The survey was conducted in the first and second months after the COVID-19 epidemic instead of during the pandemic. Hence, the variations in prevalence at other time points could not be detected. Moreover, several associated factors, such as the major of medical students, the impact of COVID-19 on students’ family, quarantine experience, and history of mental illness, have not been explored in this study. It may interfere with the accuracy of the rates of mental health problems among participants. Also, the participants were recruited from low-grade medical students at Central South University. It is incapable to represent the medical students across the whole country. Therefore, future investigations should recruit medical students from different schools and different regions across the country to constitute a more representative sample.

Overall, the study investigated the current status of mental health and cognitive function in medical students. We analyzed the correlation between sociodemographic characteristics and mental health and cognitive function in medical students and speculated on the potential factors that may induce the significant difference in each group. These findings will contribute to confirming the high-risk populations and providing target interventions to support medical students who suffered various mental and cognitive dysfunctions post-COVID-19 pandemic.

## Data availability statement

The original contributions presented in the study are included in the article/supplementary material, further inquiries can be directed to the corresponding author.

## Ethics statement

The studies involving human participants were reviewed and approved by the Ethics Committee of the Second Xiangya Hospital of Central South University (approval number: 047). The patients/participants provided their written informed consent to participate in this study.

## Author contributions

JC, ML, ZH, BW, YZ, and BL designed the study and wrote the protocols. JC, ML, JL, YJ, RX, BL, and BW participated in the data collection and organization. JC and ZH undertook the statistical analysis and wrote the manuscript, and then all authors participated in the revision. All authors are accountable for all aspects of the work in ensuring that questions related to the accuracy or integrity of any part of the work are appropriately investigated and resolved.

## Conflict of interest

The authors declare that the research was conducted in the absence of any commercial or financial relationships that could be construed as a potential conflict of interest.

## Publisher’s note

All claims expressed in this article are solely those of the authors and do not necessarily represent those of their affiliated organizations, or those of the publisher, the editors and the reviewers. Any product that may be evaluated in this article, or claim that may be made by its manufacturer, is not guaranteed or endorsed by the publisher.
